# *DLX5, FGF8* and the *Pin1* isomerase control ΔNp63α protein stability during limb development: a regulatory loop at the basis of the SHFM and EEC congenital malformations

**DOI:** 10.1093/hmg/ddu096

**Published:** 2014-02-25

**Authors:** Michela Restelli, Teresa Lopardo, Nadia Lo Iacono, Giulia Garaffo, Daniele Conte, Alessandra Rustighi, Marco Napoli, Giannino Del Sal, David Perez-Morga, Antonio Costanzo, Giorgio Roberto Merlo, Luisa Guerrini

**Affiliations:** 1 Department of Biosciences, University of Milano, Milano I-20133, Italy; 2 Telethon Laboratory, Department of Molecular Biotechnologies and Health Sciences, University of Torino, Torino I-10126, Italy; 3 Molecular Oncology Unit, LNCIB Area Science Park, Trieste I-34149, Italy; 4 Department of Biochemistry and Molecular Biology, Center for Genetics & Genomics, and Center for Stem Cell & Developmental Biology, MD Anderson, Houston, TX, USA; 5 Laboratoire de Parasitologie Moléculaire, IBMM-DBM, Université Libre de Bruxelles, Gosselies B-6041, Belgium and; 6 Department of Dermatology, University of Rome ‘Tor Vergata’, Rome I-00133, Italy

## Abstract

Ectrodactyly, or Split-Hand/Foot Malformation (SHFM), is a congenital condition characterized by the loss of central rays of hands and feet. The p63 and the *DLX5;DLX6* transcription factors, expressed in the embryonic limb buds and ectoderm, are disease genes for these conditions. Mutations of *p63* also cause the ectodermal dysplasia–ectrodactyly–cleft lip/palate (EEC) syndrome, comprising SHFM. Ectrodactyly is linked to defects of the apical ectodermal ridge (AER) of the developing limb buds. FGF8 is the key signaling molecule in this process, able to direct proximo-distal growth and patterning of the skeletal primordial of the limbs. In the limb buds of both *p63* and *Dlx5*;*Dlx6* murine models of SHFM, the AER is poorly stratified and *FGF8* expression is severely reduced. We show here that the *FGF8* locus is a downstream target of DLX5 and that FGF8 counteracts Pin1–ΔNp63α interaction. *In vivo*, lack of *Pin1* leads to accumulation of the p63 protein in the embryonic limbs and ectoderm. We show also that ΔNp63α protein stability is negatively regulated by the interaction with the prolyl-isomerase Pin1, via proteasome-mediated degradation; p63 mutant proteins associated with SHFM or EEC syndromes are resistant to Pin1 action. Thus, DLX5, p63, Pin1 and FGF8 participate to the same time- and location-restricted regulatory loop essential for AER stratification, hence for normal patterning and skeletal morphogenesis of the limb buds. These results shed new light on the molecular mechanisms at the basis of the SHFM and EEC limb malformations.

## INTRODUCTION

The *p63* gene codes for a transcription factor related to the *p53* and *p73* tumor suppressor genes, proposed as a master regulator of epidermal stem cell maintenance and proliferation, able to promote the epithelial stratification program typical of the mammalian skin. To date, several mutations in the *p63* gene have been identified associated with distinct human developmental syndromes, characterized by common features such as limb abnormalities, ectodermal dysplasia, and facial clefts (1–4). These syndromes are: the ectodermal dysplasia–ectrodactyly–cleft palate (EEC, MIM #129900), the ankyloblepharon–ectodermal dysplasia–clefting (AEC, MIM #106260), the limb–mammary syndrome (LMS, MIM #603543), the acro–dermato–ungual–lacrimal–tooth (ADULT, MIM #103285) and non-syndromic split-hand/foot malformation type-4 (SHFM-IV, MIM #605289) (1–4).

*p63* homozygous mutant mice show severe defects affecting their limbs, skin and craniofacial skeleton (5–7). In *p63^−/−^* newborn animals, the hindlimbs (HLs) are absent whereas the forelimbs (FLs) are severely truncated in their distal segment. The limb defects of *p63^−/−^* mice have been associated with failure of stratification and signaling of the cells of the apical ectodermal ridge (AER), a transitory specialization of the ectoderm at the dorsal–ventral border of the limb bud, essential for proximo-distal growth of the limbs and patterning of the fingers (8–10). *p63* is expected to control AER function and maintenance via transcriptional regulation of AER-restricted target genes (2,11,12). Failure of AER stratification has also been associated with loss of expression of key morphogens for limb development, such as *FGF8* and *Dlx* genes (2).

Within the EEC disease phenotype, ectrodactyly (also known as SHFM, MIM #183600) is a recurrent finding and consists in the absence of the distal portion of the central rays of upper and lower limbs, resulting in a deep medial cleft, missing or hypoplastic central fingers and fusion of the lateral ones. In addition to be part of the EEC syndrome, SHFM comprises both sporadic and hereditary forms, syndromic or isolated, linked to six distinct loci (types I–VI) (2,13–15). The most common form, SHFM type-I, is associated with deletions of variable extent on chromosome 7q21, the minimal common deletion includes *DSS1* and the homeogenes *DLX5* and *DLX6* (16,17). Recently, a point mutation in the DNA-binding domain of *DLX5* (Q178P) has been reported in a SHFM-I family with a recessive transmission, co-segregating with the limb malformations (18). In the mouse, the double knock-out (DKO) of *Dlx5* and *Dlx6* leads to an ectrodactyly phenotype affecting the HLs (19,20), fully confirming that the human orthologs *DLX5* and *DLX6* are the disease genes for this malformation.

SHFM type-IV (MIM #605289) is caused by mutations in the *p63* gene. In 50 unrelated patients with isolated SHFM, 5 mutations in *p63* were found, suggesting that these may account for ∼10% of sporadic cases of SHFM (1,4,21). Finally, SHFM type-III (MIM #246560) is linked to abnormalities of a genomic region comprising *dactylin* and several other genes, in mice and man; however, no disease gene has convincingly been demonstrated, as to date (22–24). Notably, the *FGF8* locus resides in the SHFM-III region; thus, this gene may represent a valid candidate for SHFM type-III (2).

Several studies have attempted to define p63-dependent transcription regulatory networks (25,26) with the hope to identify core genes and regulation at the basis of normal ectoderm development and differentiation, as well as to provide clues on the molecular bases of the ectodermal phenotypes in the EEC. Specifically, ectrodactyly has been linked to the ability of p63 to regulate transcription of *Dlx5* and *Dlx6*, both *in vitro* and in the developing embryonic limbs (7). This regulation takes place both at the proximal promoter level and via a conserved *cis*-acting genomic element, located ∼250 kb centromeric to *DLX5*, that is deleted in a family with SHFM type-I (25). Thus, the *Dlx5* and *Dlx6* genes are true p63 transcriptional targets, whose regulation during limb development is presumably needed to maintain the specialization and stratification of the AER cells (2,7).

While the pathways downstream of p63 are beginning to be elucidated, our knowledge on the upstream regulations of p63 is minimal. The main questions that arise are: how is p63 expression maintained in (proliferating) ectodermal stem cells? How is p63 down-modulated in differentiating cells? How are changes in p63 level linked to loss of AER stratification and the onset of the SHFM phenotypes? Recently, one such regulation has been identified and consists in a loop-like regulation between p63 and IRF6 (27). Several biochemical observations suggest that the ΔN- and TA-p63 proteins are tightly regulated at post-translational level, via protein modification (phosphorylation, sumoylation and ubiquitination) and protein–protein interactions (28–30).

Here we show that the prolyl *cis/trans* isomerase Pin1 acts an additional regulator of p63 protein stability, inducing a phosphorylation-dependent, proteasome-mediated degradation of wild-type (WT) ΔNp63α, but not of a disease-causing p63 mutant. Conversely, FGF8 appears to counteract Pin1-induced p63 degradation, and thus to promote p63 stability by inhibiting the interaction of ΔNp63α with Pin1, *in vivo*. As the *FGF8* locus appears to be regulated by both p63 and DLX5, we propose a model in which these two SHFM-causing genes and *FGF8* take part in a regulatory loop that opposes Pin1-mediated degradation of p63, hence permitting stratification and specialization of ectoderm cells into the AER, in a time and region-restricted manner during limb development. In SHFM type-I, type-III and type-IV, such regulation is impaired leading to reduced AER stratification, limb malformation and skeletal defects.

## RESULTS

### The AER of *Dlx5;Dlx6* DKO embryos is poorly stratified

Mutations in *DLX5* or complex genomic alterations around the *DLX5;DLX6* locus cause, in human, the malformation known as SHFM type-I (16,18). In mice, the combined deletion of *Dlx5* and *Dlx6* (*Dlx5;Dlx6* DKO) causes a limb phenotype identical to human SHFM-I and is accompanied by reduced expression of *FGF8* in the AER cells (19,20). Thus, the *Dlx5;Dlx6* DKO mice represent a valid animal model for SHFM-I, and we set forth to use them to examine whether the loss of *Dlx5;Dlx6* may result in altered AER stratification.

**Figure 1.**
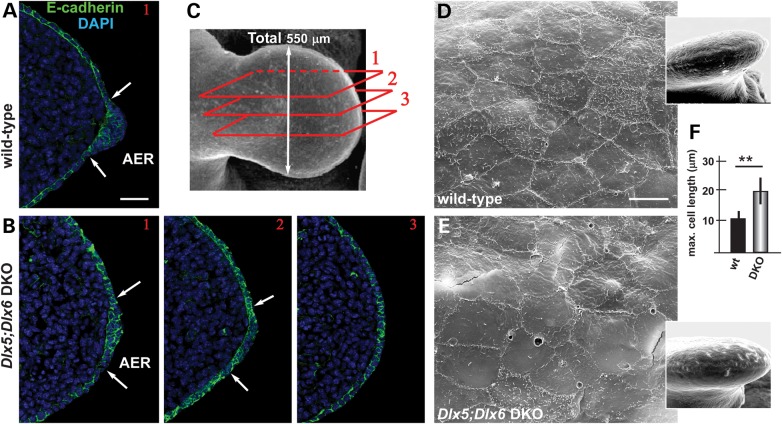
Impaired AER stratification in the limbs of *Dlx5;Dlx6* DKO mutants. (**A**–**C**) Immunofluorescent staining on WT and *Dlx5;Dlx6* DKO HLs, to detect E-cadherin (green), on serial transverse sections of the limbs, at E11.5. White arrows indicate the extension of the AER ectoderm. The position of the section planes of the micrographs (in A and B) along the anterior-to-posterior (1, 2, 3) are show in C. Scale bar in A = 20 μm. (**D**,**E**) SEM of the surface of the AER cells of WT (D) and *Dlx5;Dlx6* DKO (E) limbs. The mutant AER cells appear larger, show fuzzy borders and nearly lack microvilli. Scale bar in D = 10 μm. (**F**) Quantification of the size of AER cells (maximum cell length) comparing WT versus *Dlx5;Dlx6* DKO mutant specimens. The WT was used for normalization and made to 1. *P* < 0.02.

We stained sections of WT and *Dlx5;Dlx6* DKO HL, at the ages E11.5 and E12.5, with anti-E-cadherin antibody and examined the stratification of the AER cells. We specifically focused on the central wedge of the AER, because previous publications have indicated that only this wedge loses expression of *FGF8*, *Bmp4*, *Msx2* and *Dlx2* (2,19,20,31 and our unpublished data). At earlier ages (E11.5), the organization of the *Dlx5;Dlx6* mutant AER appeared very similar to the WT (not shown), whereas at later ages (E12.5), the central wedge of the *Dlx5;Dlx6* mutant AER appeared less stratified compared with the equivalent region of the normal limbs (sectors 2 and 3, Fig. 1B). Notably, in more lateral regions of the AER (sector 1), the stratification was normal.

To further document this finding, we carried out scanning electron microscopy (SEM) on the AER of normal and *Dlx5;Dlx6* DKO limbs at the age E12.5 and observed that the cells of the central wedge of the *Dlx5;Dlx6* mutant AER appeared morphologically abnormal, with an increase in the length of the maximum diameter (10.8 ± 2 versus 19.7 ± 4.3; *P* < 0.03), more irregular borders and fewer microvilli on the apical surface (compare Fig. 1E with D).

Thus, between E11.5 and E12.5, in the absence of *Dlx5* and *Dlx6*, the central wedge of the AER fails to specialize into a pluristratified epithelium. Notably, we and others have previously shown that the AER of p63-null and of p63-R279H homozygous mutant limbs is poorly stratified, and this is accompanied by reduced *FGF8* expression and the appearance of severe limb defects (5–7).

### The AER of *Dlx5;Dlx6* DKO limbs shows reduced levels of ΔNp63α

**Figure 2.**
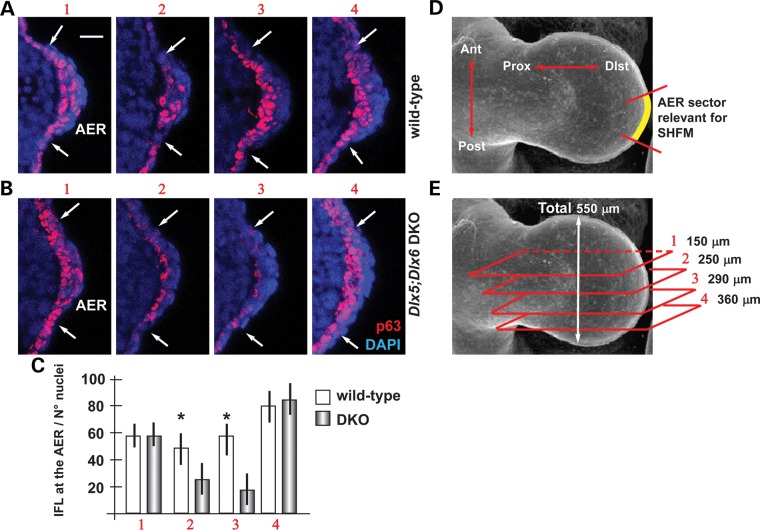
p63 protein level is reduced in *Dlx5;Dlx6* mutant AER cells. Immunofluorescent detection of p63 in the nuclei of the AER of WT and *Dlx5;Dlx6* DKO limbs, at the age E11.5. (**A**,**B**) Micrographs of WT (panels in A) and *Dlx5;Dlx6* mutant (panels in B) sections, corresponding to the positions (1, 2, 3 and 4) indicated in **E**. White arrows indicate the extension of the AER. Scale bar in A = 20 μm. (**C**) Semi-quantitative assessment of p63 signal in the AER of WT (open bars) and *Dlx5;Dlx6* mutant (gray bars), expressed as intensity per 100 nuclei, in arbitrary units. The results show that p63 staining is significantly reduced (−50%) in the positions 2 and 3 of the AER of the mutant limbs, relative to the WT. (**D**,E) Schemes illustrating the wedge of the AER relevant for the SHFM phenotype (yellow in D) and the position of the section planes (1, 2, 3 and 4) along the anterior-to-posterior axis (red rectangles in E). The numbers on the left of each section plane indicate the distance from the anterior margin.

We decided to further investigate the molecular link connecting *Dlx5;Dlx6* and p63 in the embryonic limbs. We previously established that the expression of *ΔNp63* and *TAp63* mRNAs is not significantly changed in the *Dlx5;Dlx6* DKO limbs (7), and we also excluded changes in *Pin1* mRNA or protein levels (Supplementary Material, Fig. S1). Thus, we ruled out a direct transcriptional regulation for the observed reduction of p63 in the *Dlx5;Dlx6* DKO limb buds (Fig. 2) and opted for a post-transcriptional type of regulation.

Considering that p63 is essential for stratification of ectoderm-derived epithelia, we set forth to determine whether the absence of *Dlx5;Dlx6* may lead to altered levels of p63 protein in the AER cells of the developing limb buds. We carried out immunostaining for p63 on serial sections of the HLs, focusing on the central wedge of the AER, and semi-quantified the signal intensity along the anterior-to-posterior length of the limb bud (schemes in Fig. 2D and E). In the AER nuclei of the central wedge (sectors 2 and 3, Fig. 2B), p63 immunostaining is significantly reduced as compared with the same region of the normal limb (sector 2, −50%; sector 3, −65%), whereas no such difference was observed in lateral wedges of the AER (sector 1) or in the non-AER ectoderm (Fig. 2A and B, quantification in C).

Thus, p63 is down-regulated in the central AER cells by post-transcriptional mechanisms, at the same time as these cells fail to efficiently stratify. As p63 has been directly linked to ectodermal stratification (11), we can hypothesize that the mis-organization of the AER cells in *Dlx5;Dlx6* DKO limbs might be the consequence of altered p63 levels.

### Pin1 interacts with ΔNp63α and promotes its destabilization

The stability of the p63 protein is tightly regulated by the action of several interacting or modifying proteins, including MDM2 and p53 (28–30). The enzyme peptidyl-prolyl *cis/trans* isomerase NIMA-interacting-1, Pin1, has been shown to modulate the activity of p53 and p73 by post-translational modifications (32–36). We therefore examined the possibility that Pin1 may also regulate ΔNp63α protein level and consequently modulate its activity, by a similar mechanism. Notably, previous experiments have shown a diminished transcription of two p63 targets in Pin1-overexpressing cells *in vitro* (37), suggesting that Pin1 could modulate p63 protein levels and/or activities.

**Figure 3.**
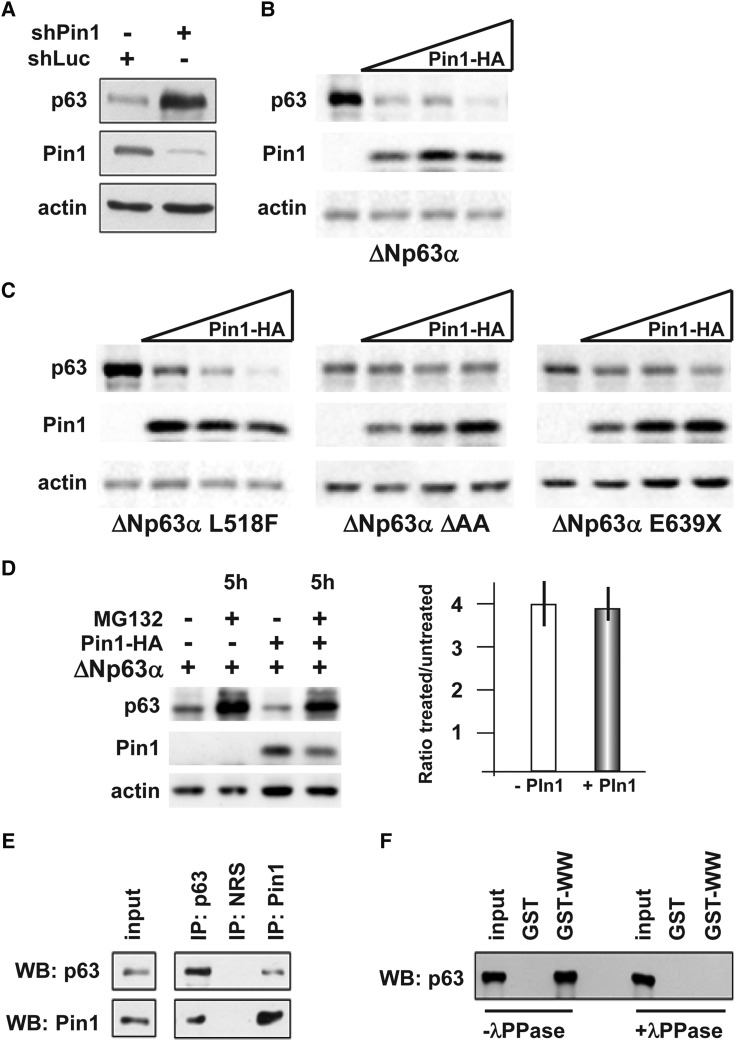
The Pin1 isomerase promotes ΔNp63α degradation. (**A**) Western blot analyses of whole protein extracts of U2OS cells transiently co-transfected with ΔNp63α (50 ng) and an anti-Pin1 siRNA (10 pmol/cm^2^), or an anti-LacZ siRNA (siCtr 10 pmol/cm^2^) as negative control. Actin is used for loading control. (**B**) Western blot analyses of whole protein extracts from U2OS cells transiently co-transfected with increasing amounts (20, 40 and 80 ng) of Pin1–HA-tagged vector (indicated on top), and WT ΔNp63α (30 ng). The α-isoform is efficiently degraded by Pin1 expression, whereas the β- and the γ-isoforms are not (Supplementary Material, Fig. S1). (**C**) Western blot analyses of whole protein extracts from U2OS cells transiently co-transfected with increasing amounts (20, 40 and 80 ng) of Pin1–HA-tagged vector (indicated on top), and the disease-relevant mutated versions of ΔNp63α L518F (AEC associated), ΔAA (LMS associated) and E639X (SHFM associated) (30 ng each) (indicated at the bottom). Actin is used for loading control. The mutant p63 proteins linked to congenital limb malformations (LMS and SHFM) are relatively resistant against Pin1-induced degradation, compared with WT p63, whereas the mutant p63 linked to AEC, with no limb defects, is sensitive to Pin1. (**D**) Western blot analyses of whole protein extracts from U2OS cells, transiently co-transfected with ΔN-p63α and Pin1–HA-tagged expression vectors, and 20 h later either treated with 5 μm of the proteasome inhibitor MG132 or left untreated (DMSO only). Proteins were extracted after 5 h of treatment and assayed by western analysis; actin is used for loading control. On the right, quantification of the p63 protein level, expressed as the ratio between the treated and the untreated sample, in cell transfected (gray bar) or not transfected (open bar) with Pin1. The results show that Pin1-mediated destabilization of p63 is mainly mediated by the proteasome. (**E**) Western blot analyses of proteins immunoprecipitated with anti-p63 (IP p63) or anti-Pin1 (IP Pin1) antibodies, revealed using, respectively, anti-Pin1 (bottom, lanes) and anti-p63 (top panel). Input control is shown on the left. (**F**) Western blot analysis of GST-pull down assay done with anti-Pin1, revealed with anti-p63 antibody, in the presence (+λPPase, on the right) or absence (−λPPase, on the left) of protein phosphatase during the pull-down. The input sample is also loaded as control. While untreated samples contained p63, in the presence of IPP the p63 protein is absent.

To verify this point, we adopted an siRNA-based approach to down-regulate endogenous Pin1 expression in the U2OS human osteosarcoma cell line, which does not express *p63* endogenously. We transfected the U2OS cells with ΔNp63α and an anti-Pin1 siRNA. The depletion of Pin1 resulted in a significant stabilization of ΔNp63α, as compared with control-silenced cells (Fig. 3A). Conversely, overexpression of Pin1 resulted in a marked and dose-dependent reduction of ΔNp63α protein levels (Fig. 3B). The same experiment was performed on the A431 human epidermoid squamous carcinoma cell line, which expresses ΔNp63α endogenously and yielded similar results (data not shown).

We then tested whether disease-causing mutant p63 proteins are sensitive to the degrading action of Pin1, by transfecting expression vectors carrying the L518F (linked to AEC syndrome), the ΔAA (linked to LMS syndrome) or the E639X (linked to SHFM-IV syndrome) point-mutated variants. While the AEC mutant p63 protein was still sensitive to Pin1-induced degradation, the LMS and SHFM mutants were more resistant to such effect (Fig. 3C). Interestingly, while the AEC syndrome is not associated with limb developmental defects, the LMS and SHFM syndromes typically entail an ectrodactyly phenotype.

We then tested whether the effect of Pin1 on p63 might be mediated by the proteasome. U2OS cells were co-transfected with a Pin1 and a ΔNp63α expression vectors and then treated with the proteasome inhibitor MG132; we observed that the Pin1-induced depletion of ΔNp63α was partially reversed in MG132-treated cells, compared with controls (Fig. 3D), suggesting that Pin1-induced p63 protein destabilization is in part mediated by the proteasome.

We then examined whether Pin1 and p63 proteins physically interact *in vivo*. We carried out co-ImmunoPrecipitation (co-IP) experiments in HaCaT cells, which express both proteins endogenously, using anti-Pin1 and anti-p63 antibodies. co-IP with the anti-p63 antibody was able to pull down Pin1, and vice versa, indicating that these two proteins interact, either directly or indirectly via complex formation (Fig. 3E). Pin1 is known to interact with its partner proteins in a phosphorylation-dependent manner and to catalyze *cis/trans* isomerization of selected peptide bonds (38). Therefore, we tested whether the Pin1–p63 interaction might depend on phosphorylation. We transfected the U2OS cells with ΔNp63α and then detected the interacting proteins by using GST-WW (or GST as a control), in the presence or absence of λ-phosphatase, as previously described (39). Treatment of the cell lysates with λ-phosphatase resulted in a loss of the interaction between Pin1 and p63 (Fig. 3F), suggesting that this interaction requires a phosphorylation event.

### Absence of *Pin1* results in increased levels of ΔNp63α protein *in vivo*

**Figure 4.**
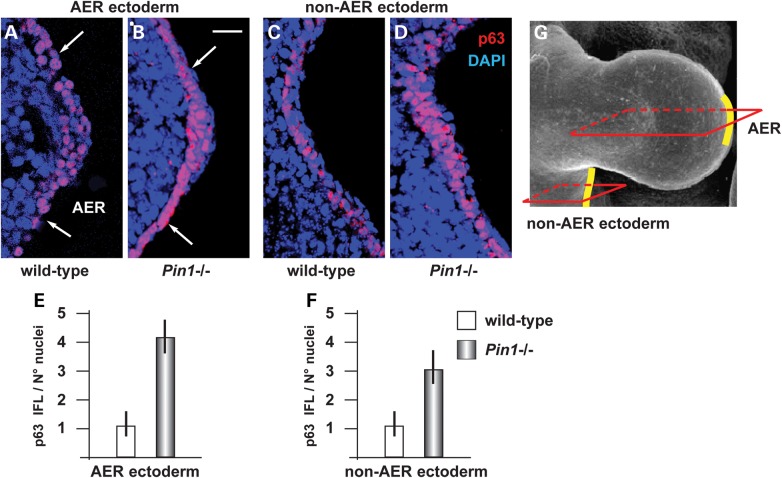
Loss of Pin1 causes stabilization of p63 in the embryonic ectoderm. (**A**–**D**) Immunofluorescent detection of p63 on the AER ectoderm (A,B) and the non-AER ectoderm (C,D) of WT (A,C) and Pin1 KO (B,D) embryonic limbs, at the age E11.5. White arrows in A–C indicate the extension of the AER ectoderm. IFL signal is specifically detected in the nuclei. (E,F) Semi-quantitative assessment of p63 signal in the AER (**E**) and the non-AER ectoderm (**F**), expressed as intensity per 100 nuclei, in arbitrary units. Wild-type was normalized and made to 1. In the absence of Pin1, the p63 signal is increased ∼4-folds in the AER, and ∼3-folds in the general ectoderm. (**G**) A scheme to illustrate the approximate position of the section planes in A–D, along the anterior-to-posterior axis. The AER and non-AER ectoderm are highlighted in yellow. Scale bar in B = 20 μm.

We next sought evidence that Pin1 regulates p63 protein levels during embryonic development, and specifically in the embryonic ectoderm. We collected samples of ectoderm and limb buds from E11.5 WT and *Pin1*^−/−^ embryos (40,41) and stained sections with an anti-p63 antibody that recognizes all p63 isoforms. Of note, the ΔNp63α isoform is the most abundantly expressed in the limb buds at this age (7). The results show that the intensity of p63 immunostaining in the nuclei was increased both in the AER (Fig. 4B) and the non-AER (Fig. 4D) ectoderm of *Pin1*^−/−^ embryos, 4- and 3-folds, respectively, relative to the WT (Fig. 4A and C).

As p63 IFL signal was found to be reduced in the *Dlx5;Dlx6* DKO limbs (Fig. 2), we asked whether this was due to increased Pin1 expression, either mRNA or proteins. Thus, we stained adjacent sections of WT and *Dlx5;Dlx6* DKO limbs with anti-Pin1 antibody, but no significant difference in the Pin1 signal was detected in the mutant limbs (Supplementary Material, Fig. S2). Antibody specificity was confirmed by complete lack of staining in *Pin1* KO embryonic limbs. We also determined the mRNA abundance of *Pin1* mRNA in RNA extracted from WT or *Dlx5;Dlx6* DKO limbs, by Real-Time qPCR, but again could not detect any significant differences (Supplementary Material, Fig. S2). Thus, we concluded that the reduced p63 levels observed in the *Dlx5;Dlx6* DKO AER cells are unlikely to be due to changes in Pin1 level.

### *FGF8* is downstream of *DLX5* and counteracts Pin1-dependent degradation of p63

We decided to further investigate the molecular link connecting *Dlx5;Dlx6* and p63 in the embryonic limbs. We previously established that the expression of *ΔNp63* and *TAp63* mRNAs is not significantly changed in the *Dlx5;Dlx6* DKO limbs (7), and we also excluded changes in *Pin1* mRNA or protein levels (Supplementary Material, Fig. S1). Thus, we excluded a direct transcriptional regulation for the observed reduction of p63 in the *Dlx5;Dlx6* DKO limb buds (Fig. 2) and opted for a post-transcriptional type of regulation.

In the AER of both *Dlx5;Dlx6* DKO and *p63* mutant limbs, expression of *FGF8* is reduced, as revealed by *in situ* hybridization (5,6,19). FGF8 is a well-known limb morphogenetic diffusible factor, essential for the maintenance of AER stratification, for limb growth and morphogenesis (42–44), and the partial or complete absence of members of the FGF family causes a set of developmental limb defects (42,45). Thus, we focused on FGF8 and raised the hypothesis that Dlx5 and/or p63 may concur to positively regulate *FGF8* transcription (2).

**Figure 5.**
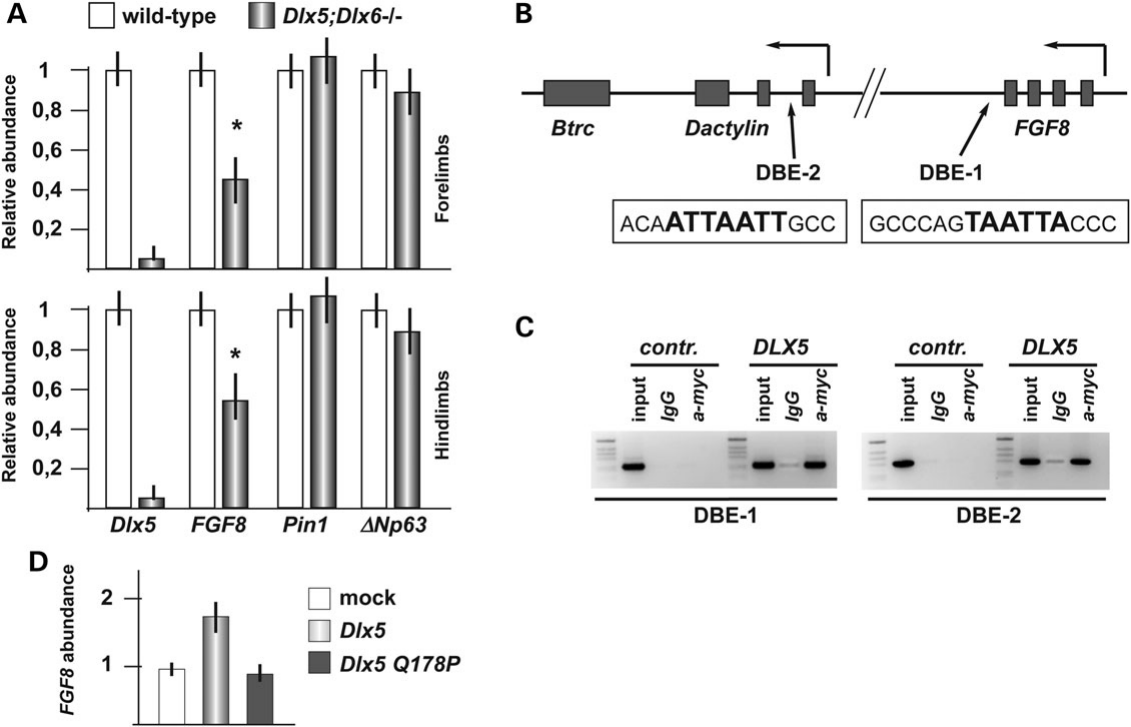
FGF8 is regulated by Dlx5. (**A**) Relative abundance of FGF8, Pin1 and ΔN-p63 mRNAs in samples from WT (gray bars) and *Dlx5;Dlx6* DKO (black bars) HLs and FLs. Values are expressed relative to the WT specimen, which was set to 1. The Dlx5 mRNA level is used to confirm the mutant genotype. Experiments were done on independent biological duplicates. (**B**) Scheme of the FGF8 and Dactylyn loci, showing the SHFM-III critical region and the approximate position of the two best predicted Dlx5 sites DBE-1 and DBE-2, indicated with black arrows (31). The exact (human or mouse) sequence corresponding to the DBE-1 and -2 sites is reported in open boxes; the bold characters represent the Dlx5 PWM (46). Solid boxes represent the exons. The position of p63 binding sites [from (25)] is reported in Supplementary Material, Fig. S3. (**C**) ChIP analysis on the chromatin of U2OS cells, transfected with DLX5-myc-tagged and immunoprecipitated with anti-myc. The DBE-1 (left) and DBE-2 (right) elements were amplified by PCR. Enrichment is detected in cells transfected with DLX5, compared with mock-transfected cells, or with chromatin precipitated with an irrelevant antibody (IgG). Input chromatin is shown of the left of each blot. (**D**) Relative abundance of endogenous FGF8 mRNA upon transfection of U2OS cells with the WT (light gray bar) or the Q178P mutant (dark gray bar) DLX5-HA expression vectors. Values are expressed relative to sample from control transfected cells (open bar), set to 1.

First, we quantified the expression of *FGF8*, *Pin1* and *ΔNp63* in the HL and FL of WT and *Dlx5;Dlx6* mutant embryos, by Real-Time qPCR, and observed that *FGF8* expression in the mutant limbs is reduced by 40%, compared with the normal limbs (Fig. 5A), whereas expression of *Pin1* and of *p63* did not significantly change. This result confirms previous *in situ* hybridization data showing reduced expression of *FGF8* in the central wedge of the AER of *Dlx5;Dlx6* DKO HLs (19) (our unpublished data). Notably, a reduction of *FGF8* mRNA abundance was also seen in the embryonic FLs of *Dlx5;Dlx6* DKO embryos, showing no evident developmental defects, suggesting that the down-modulation of *FGF8* is not the mere consequence of cell suffering, but more likely a transcriptional misregulation occurring in both the HLs and the FLs.

The *FGF8* locus lies in a genomic region that has previously been implicated in the SHFM type-III malformation in human, and to an ectrodactyly phenotype in the *dactylaplasia* mouse strain (22,23). Currently, the disease gene causing this malformation is uncertain, but there is evidence suggesting that the underlying genetic mechanism is a genomic position effect. p63 binding sites have been detected in this region, via ChIP-seq experiments on human keratinocytes (25). We searched the *FGF8* locus and flanking regions for the presence of predicted Dlx5 binding sites, conserved between mouse and human, using a position–weight matrix (PWM) approach (31,46), and detected four such sites (Fig. 5B, and Supplementary Material, Fig. S3). To verify whether DLX5 physically interacts with these genomic elements, we carried out ChIP analyses on two of these regions, named DBE-1 and DBE-2, located, respectively, ∼1 kb downstream of *FGF8* (DBE1) and within the first intron of *dactylyn* (DBE2) (Fig. 5B). We transfected U2OS cells with the *DLX5-myc-tag* expression vector, and with an empty vector as control, and then immunoprecipitated the chromatin with anti-myc-tag. The results show an enrichment of the DBE-1 and DBE-2 elements in the presence of DLX5-myc protein, compared with the negative controls (Fig. 5C).

Next we asked whether overexpression of *DLX5* in U2OS cells (not expressing *DLX5* endogenously) indeed resulted in higher *FGF8* mRNA levels. We carried out Real-Time qPCR on RNA extracted from U2OS cells transfected with either the WT *DLX5* or the Q178 mutant *DLX5* expression vectors and measured the abundance of endogenous *FGF8* mRNA. Expression of WT DLX5 resulted in a 1.7-fold increase in *FGF8* mRNA, whereas the mutant DLX5 protein had a minimal effect on FGF8 expression levels (Fig. 5D). Together, all these data indicate that *FGF8* is a transcriptional target of DLX5 and that the SHFM-linked DLX5-Q178P mutant, linked to SHFM-I, looses the capacity to efficiently activate *FGF8* expression.

**Figure 6.**
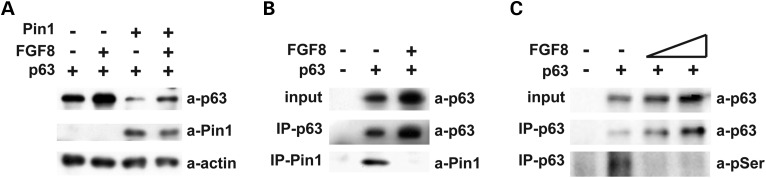
FGF8 counteracts Pin1-induced p63 degradation. (**A**) Western blot analysis of total protein extracts from U2OS cells co-transfected with the ΔNp63 expression vector (30 ng) and the Pin1–HA-tagged expression vector (40 ng) and either treated for 3 h with 1 ng/ml of recombinant FGF8 or left untreated (DMSO only). In the presence of FGF8, Pin1-induced p63 degradation is significantly less efficient. (**B**) Extract from HaCaT cells, either treated with 1 ng/ml of FGF8 or left untreated (DMSO only), immunoprecipitated with anti-p63 polyclonal antibody (p63 H-129, SantaCruz) and analyzed by western blot with either p63 monoclonal antibody or Pin1 monoclonal antibody. Input (no IP) is shown on the top. U2OS cells not expressing endogenous p63 were used as negative control. In the presence of FGF8, Pin1 is no longer able to interact and co-immunoprecipitate p63. (**C**) Extract from HaCaT cells, either treated with 1 ng/ml of FGF8 or left untreated (DMSO only), immunoprecipitated with anti-p63 and analyzed by western blot with α-phospho-ser. In the presence of FGF8, the amount of serine phosphorylation is clearly diminished.

Next we investigated by which mechanism FGF8 participates in Dlx5-Pin1-p63 regulatory loop, by investigating the effect of FGF8 on the stability/degradation of p63. We transfected U2OS cells with the *Pin1-HA-tag* expression vector and then treated the cells with either purified FGF8 or with DMSO as negative control. While in the absence of exogenous FGF8, Pin1 could efficiently down-modulate ΔNp63α protein levels, in the presence of FGF8 this effect was reduced (Fig. 6A). Similar results were obtained by treating U2OS cells with FGF2 (data not shown).

Interestingly, treatment with FGF8 alone resulted in increased levels of ΔNp63α. These results indicate that FGF8 counteracts the ability of Pin1 to induce ΔNp63α, degradation.

In order to reveal by which molecular mechanism FGF8 prevents Pin1-mediated degradation of ΔNp63α, we tested by co-IP whether FGF8 could modulate Pin1-ΔNp63α protein–protein interaction, *in vivo*. In the presence of recombinant FGF8, ΔNp63α–Pin1 interaction was significantly reduced compared with the interaction detected in the absence of FGF8 (Fig. 6B). Finally, as binding of Pin1 to its target protein is known to be dependent on phosphorylation of serine/threonine residues, we verified the phosphorylation status of ΔNp63α in untreated versus FGF8-treated cells by using anti-phospho serine and threonine antibodies on immunoprecipitated ΔNp63α from HaCaT cells. A reduction of basal serine phosphorylation levels of ΔNp63α was evident upon FGF8 treatment (Fig. 6C). Similar results were obtained with anti-phospho threonine antibodies (data not shown).

These results suggest that FGF8 protects ΔNp63α from Pin1-dependent degradation by interfering with the ability of Pin1 to physically interact with ΔNp63α.

## DISCUSSION

p63 is emerging as the master transcriptional regulator of expansion, development and differentiation of ectoderm-derived cells and tissues. Great attention has been placed on the identification of its downstream transcriptional targets (25,26,47); however, an equal complex set of regulations controls the p63 level, stability, activity, and degradation (28–30). The increasingly complex regulation upstream and downstream of p63 reflects the peculiar and critical activity of p63 to finely orchestrate the timing of exit from the cell cycle and the dynamic of stratification of mammalian ectoderm (27,48).

Ectodermal dysplasias are often accompanied by limb malformations, and specifically the p63-linked EEC comprises the ectrodactyly (SHFM) phenotype, with varying degrees of penetrance and severity. Six loci have been identified in hereditary forms of SHFM, and additional SHFM loci might exist to account for sporadic cases. For type-I and –IV, the transcription factors *DLX5-DLX6* and *p63*, respectively, are the recognized disease genes (18,49). For SHFM-III, the F-box/WD40 gene *Dactylin* has been proposed (22–24). In the recessive form SHFM type-VI, the *WNT10b* gene has recently been found mutated (15). The existence of several phenocopies of ectrodactyly has long suggested the possibility that the corresponding disease genes might participate in a regulatory cascade; however, the only established link is the transcriptional regulation of p63 on *Dlx5;Dlx6* (7,25,50). By examining the murine models of SHFM available to date, namely the *p63^null^*, *p63^EEC^* (for SHFM-IV and EEC), the *Dlx5;Dlx6* DKO (for SHFM-I) and the spontaneous mutant strain *Dactylaplasia* (*Dac*, for SHFM-III), the striking observation is that in all these models the AER shows reduced *FGF8* expression and lack or has impaired stratification, with accompanying limb developmental defects of varying severity (5–7,19,20,51).

**Figure 7.**
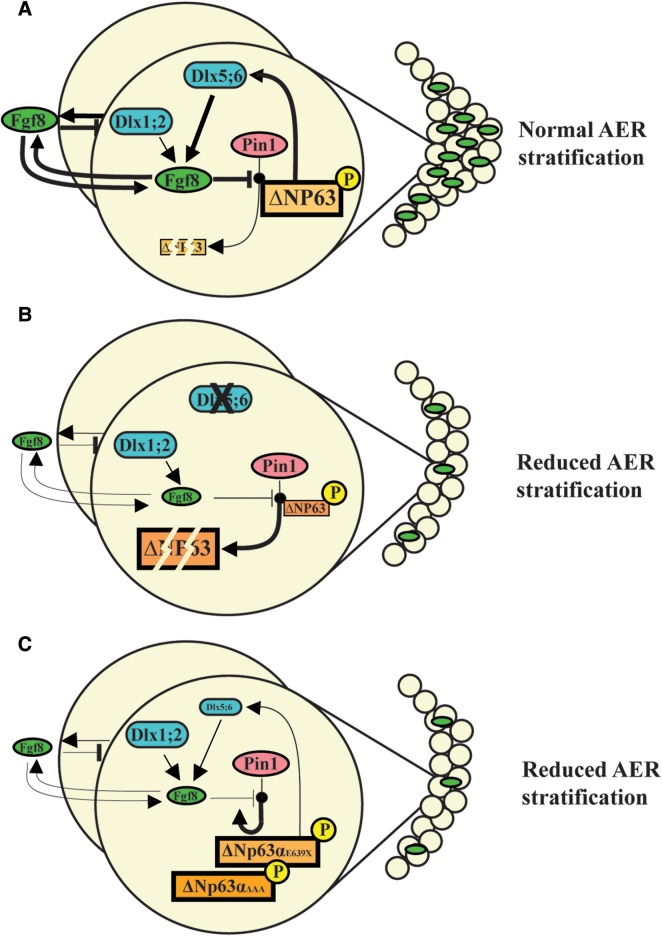
Model of the molecular loop between Dlx5 and p63 during AER stratification. Our proposed model of the regulatory loop linking the Dlx5 and the p63 SHFM-genes, via the activity of Pin1. (**A**) In WT AER cells, *Dlx5;Dlx6* positively control FGF8 transcription, a function most likely shared with Dlx1 and Dlx2 [see ref. (2)]. Likewise, p63 also activates FGF8 transcription, acting on independent genomic elements. AER-expressed FGF8 acts on the AER cells and prevent Pin1-induced ΔNp63 protein degradation: such fine mechanism dynamically maintains a control over the level of p63 in the AER cells, to assure their time- and region-restricted ability of these cells to stratify. (**B**) Mutation or loss of Dlx5 yield to a reduced FGF8 expression and an augmented ability of Pin1 to induce p63 degradation. Consequently, ΔNp63 tends to be depleted in the AER nuclei, which may result in a further down-modulation of FGF8. Reduced FGF8 and p63 cause impairment in the ability of the AER cells to stratify. (**C**) Mutant p63 associated with congenital limb malformations in human (LMS and SHFM) are relatively resistant to Pin1-induced degradation, although they appear to be transcriptionally inactive. Solid arrows indicate activation; solid stamps indicate repression. Line widths are proportional to the intensity and/or efficiency. Transcription factors are frames in squares; the other genes are framed in ovals.

Here we show that the Pin1 *cis/trans* isomerase is a regulator of p63 protein stability, inducing proteasome-mediated degradation of ΔNp63α. We also show that FGF8 counteracts this function and thus promotes p63 stability. The *FGF8* locus appears to be regulated by DLX5; thus, we propose a model (see Fig. 7) in which these two SHFM disease genes, together with *FGF8* and *FGFR1*, take part in a regulatory loop that tightly controls p63 protein level. According to our model, the activation of this loop permits stratification and specialization of ectoderm cells into the AER, in a time and region-restricted manner during limb development. In SHFM type-I, type-III and type-IV, such regulation is impaired leading to reduced AER stratification, limb malformation and skeletal defects. Although some molecular details still remain to be fully clarified, our novel findings together with previous work from our team (7) provide a developmental and molecular explanation for a set of congenital limb malformations.

Our model helps to explain other observations and findings. The *FGF8* locus is located close (∼50 kb) to the chromosomal region implicated in SHFM type-III and in the *Dac* mice. Although *Dactylyn* has been proposed as the disease gene for these malformations (22–24), no clear evidence is available on its role in limb development. Conversely, there are reasons to implicate FGF8 in the molecular pathogenesis of this disorder. First, the SHFM-III/Dac rearrangement does not interrupt any gene, and therefore, it is likely to act by perturbing the chromosomal organization and affecting expression of nearby genes; second, the *FGF8* expression is reduced in the limb buds of *Dlx5;Dlx6*, *p63^null^*, *p63^EEC^* and *Dac* mutant embryos (5–7,19,22) (and this report); third, the presence of Dlx5 (this report) and p63 (25) binding sites in conserved genomic regions near the *FGF8* and the *Dactylyn* loci; fourth, FGF8 plays a critical role for limb bud growth, patterning, morphogenesis as well as AER maintenance (9,42,51–53). Thus, it is tempting to propose that misregulation of *FGF8* expression is the molecular lesion at the basis of SHFM-III/Dac; however, direct evidence for this is lacking and should be explored in future works.

The role of FGF8 in the signaling from the AER for the proximal limb development is well known (45,52), however not fully comprehended in cellular terms. The AER-specific conditional disruption of *FGF8* does not lead to altered AER induction and stratification, *per se* (54); however, this could be explained by the fact that during limb development, FGF4, FGF9 and FGF17 have been shown to compensate for the loss of *FGF8* (45). Conversely, the AER-specific conditional disruption of *Fgfr2* leads to altered AER stratification and function and limb defects (55). AER-derived FGFs have been shown to promote non-directional mesenchymal cell movements during limb bud morphogenesis (44), and consistently, the conditional inactivation of *Fgfr1* in the limb mesoderm disrupts the relative proportions of the limb elements and leads to profound limb malformations (56). Most relevant, mutations in *FGFR1* have recently been found in patients with Hartsfield syndrome (OMIM 615465), a congenital condition comprising ectrodactyly (57). This finding clearly supports the notion that impairment of the FGF signaling is directly involved in the molecular pathogenesis of ectrodactyly.

While FGFs promote mesenchymal cell movements, in the same article the authors show that Wnt5a promotes oriented cell divisions/movements during limb development (44), and Wnt5a is a known target of Dlx5 (58). We believe that these findings are highly relevant for the comprehension of the SHFM malformation, and we are tempted to speculate that the reduced expression of *FGF8* and *Wnt5a* (our unpublished data) in the central AER of *Dlx5;Dlx6* DKO limbs may induce mis-oriented divisions/movements of mesenchymal cells in this sector, hence altered morphogenesis/loss of central digits. This possibility warrants future experimental work. When a more complete model will be available, the hope is to be able to exploit this knowledge to restore normal levels of these soluble signaling factors, toward correcting the SHFM defects.

Altered Pin1-dependent p63 regulation may impact on several cellular processes, in addition to ectoderm stratification. Cells from *Pin1* knock-out mice have difficulties in exiting the G0 and entering the S phase, and *Pin1* null animals have meiotic defects and are hypofertile (40,41). In these animals, an altered phosphorylation levels of RB correlate with tumor growth (59). However, as Pin1 interacts with p53 and p73, the contribution of p63 is uncertain. Likewise, a Pin1/mutant p53 axis has been identified that promotes aggressiveness of breast cancer cells; however, the relevance of p63 in this context is not well defined (37). A role for p63 has been established in cancer types of ectodermal or endodermal origin, in particular lung and skin carcinomas. Indeed, ΔNp63α regulates keratinocyte proliferation by controlling PTEN expression and localization (60). Notably, a mis-activation of p63 in squamous cell carcinomas has been functionally linked with the activation of the FGFR2 receptor (61), further supporting the view that FGFs participate in Pin1-dependent p63 stability.

It would be important to define whether this regulatory pathway participates in skin carcinogenesis.

## MATERIALS AND METHODS

### Mouse strains

The *Dlx5;Dlx6* DKO mouse strain (20) was maintained in a mixed C57/BL6:DBA genetic background. The *Pin1* null mouse strain was originally generated by Fujimori and coworkers (40), then transferred onto a C57/BL6 pure background by Atchison and coworkers (41) and maintained in this background. The day of the vaginal plug was considered as embryonic age 0.5. Embryos were collected at the indicated ages in cold PBS, fixed in cold 4% PFA for 8–12 h and processed for cryopreservation and sectioning according to standard protocols. Extra-embryonic tissues were used for genotyping by PCR.

### Immunofluorescence on embryonic limbs

Longitudinal sections of 12–15 μm were collected on glass slides, blocked with PBS with 1% BSA for 1 h at RT and incubated with the following primary antibodies, diluted from 1 : 250 to 1 : 50 in PBS + 1% BSA, ON at 4°C: anti-Pin1 (G8 sc-46660, Santa Cruz), with anti-p63 (4A4 sc-8431, Santa Cruz) and with anti-E-cadherin (36/E 610182, BD) and then incubated with secondary antibodies anti-mouse-Cy2 and anti-rabbit-Cy3 (Jackson ImmunoResearch) diluted 1 : 200, 1 h at RT, washed, stained with DAPI for the nuclei detection and examined with a Zeiss Observer-Z1 fluorescent microscope, equipped with Apotome system. Raw images were digitally processed to normalize the background and optimize the contrast, with Photoshop (Adobe), and mounted with QuarkXpress (Pantone).

Semi-quantitative immunofluorescence analysis was performed with ImageJ-64 (v1.45) software. Images were first converted to grayscale, and the DAPI channel was used to count nuclei. p63 intensity was quantified after background correction and normalized respect to the number of nuclei in the region of interest. Data are presented as mean and s.d. of ∼4/5 different sections of three different embryos. A significant *T*-test score is indicated by asterisks: *indicates *P* < 0.05, **indicates *P* < 0.01.

### Plasmids

Vectors expressing the WT ΔNp63α isoform of, or the disease-linked mutant *p63*, were previously described (62,63). The *DLX5-*myc-tagged expression vectors were obtained from OriGene, and previously used (58). The Q178P *DLX5-myc* point mutation [based on the sequence in NM_005221.5 (18)] was generated by site-directed mutagenesis in the *DLX5-myc* expression plasmid and sequence-verified (Bio-Fab Research, Rome, Italy). The *Pin1* si-RNA was previously described (37).

### Cell cultures and transfections

The U2OS human osteosarcoma and the A431 human epidermoid squamous carcinoma cell lines were maintained in Dulbecco's modified Eagle's medium (D-MEM) and 10% fetal bovine serum. For transfection, 50,000 cells were seeded into 24-well multi-plates and the next day transfected with Lipofectamine 2000 (Invitrogen) according to the manufacturer's instructions. The total amount of transfected DNA (500 ng) was kept constant using empty vector when necessary. After 24 h, cells were lysed and assayed for western blot analysis (29). MG132 treatment was initiated the day after transfection with 5 μm MG132 (Sigma) for 5 h. FGFs treatments were initiated 20 h after transfection with 1 ng/ml of FGF8 or FGF2 for 3 h.

### Western blot analyses

Twenty-four hours after transfection, cells were lysed in 100 μl of loading buffer (2% sodium dodecyl sulfate, 30% glycerol, 144 mm β-mercaptoethanol, 100 mm Tris–HCl pH 6.8 and 0.1% Bromo-Phenol Blue): extracts were separated on SDS–10% polyacrylamide gel, transferred on nitrocellulose membrane (Protran, Millipore) and incubated with the relative antibodies and developed according to the manufacturer's instructions (GeneSpin). The following primary antibodies were used: α-p63 (4A4, sc- 8431, Santa Cruz), α-actin mouse monoclonal (A2066, Sigma), α-Pin1 mouse monoclonal (G9, sc-46660, Santa Cruz), α-Pin1 rabbit polyclonal, α-phospho-ser polyclonal (Invitrogen, 618100) and α phospho-thr polyclonal (Cell Signaling, # 9381). As secondary antibodies, we used the following: α-mouse secondary (sc-2005, Santa Cruz) and α-rabbit secondary (sc-2030, Santa Cruz).

### Co-immunoprecipitation

HaCaT cells (4 × 10^6^/150 mm plate) were treated with FGF8 (1 ng/ml) for 3 h and then harvested for the preparation of whole-cell lysates using RIPA buffer [10 mm Tris–HCl pH 8, 2 mm EDTA, 0.1% SDS, 0.1% sodium deoxycholate, 140 mm NaCl, 1× Triton, supplemented with 1 mm phenylmethylsulfonyl fluoride and protease inhibitors (all from Sigma)]. Cell lysates were incubated on ice for 20 min., vortexed and centrifuged at 6600×*g* for 10 min to remove cell debris. Protein concentration was determined with Bradford Reagent (Sigma). Three milligrams of cell lysate was incubated overnight at 4°C with 3 μg of anti-p63 (H-129, sc-8344, SantaCruz). The immunocomplexes were collected by incubating with a mix of Protein A–Agarose and Protein G–Sepharose (Sigma) overnight at 4°C. The beads were washed three times: the first wash with RIPA buffer and the others with PBS. The beads were then resuspended in 2× Lysis buffer, loaded directly on a 10% SDS–polyacrylamide gel and subjected to western blot with the indicated antibodies.

### mRNA quantification by Real-Time qPCR

Embryonic FLs and HLs were dissected from embryos at the indicated age, in cold RNase-free PBS, under microscopic examination. A minimum of three limbs were pooled, according to the genotype and collected in Trizol (Invitrogen). Total RNA was extracted with the TRI reagent (Sigma) and treated with DNAse I (Ambion). Reverse-transcription and cDNA synthesis were done using kits (Invitrogen), as previously reported (7,64). Three nanograms of each cDNA sample were used in Real-Time qPCR analyses using SYBR green IQ reagent (Biorad) on T900 HT Fast Real Time PCR Sistem (Applied Biosystems). The *TATA-binding protein* (*TBP*) and the *GAPDH* mRNAs were used for normalization. Primer sequences are provided in Supplementary Material, Table S1. Experiments were repeated twice on independent samples; every point was done on biological duplicates. Analyses were performed with ABI 2.1 software (Applied Biosystems).

Total RNA from U2OS cells was extracted using the TRI reagent and treated with DNase-I (Ambion). One micrograms of RNA was retrotranscribed with SuperScriptII (Invitrogen). qPCR was performed using SYBR green IQ reagent (Biorad) on the Rotor Gene machine. Primers were designed to amplify regions of 80–120 bp in size. *Tubulin* and *GAPDH* mRNAs were used for normalization. Experiments were repeated twice on independent samples. Primer sequences are provided in Supplementary Material, Table S1.

### Chromatin immunoprecipitation

ChIP analyses were performed on sheared genomic DNA from 1 × 10^6^ U2OS cells transfected with 12 μg of *DLX5-myc* or *DLX5-Q178P-myc* vectors, or with the empty pcDNA3 vector as control and immunoprecipitated with 5 μg of anti-myc-TAG mouse monoclonal antibody (SantaCruz, sc-40) or 5 μg of anti-Flag antibody (Sigma, F3165) as previously described (7). For negative control, an irrelevant antibody was used. The sequences of the oligonucleotides used for this analysis are provided in Supplementary Material, Table S1).

## SUPPLEMENTARY MATERIAL

Supplementary Material is available at *HMG* online.

## FUNDING

This work was supported by grants from the Italian Telethon Foundation (GGP11097 to L.G., A.C. and M.G.R.), from Fondazione Ricerca Biomedica (to M.G.R.), from Ministero Italiano Università e Ricerca (PRIN2009 to A.C. and L.G.; RBAP10XKNC-003 and PRIN 2009–2009YP9AE5 to G.D.S.) and from AIRC Special Program Molecular Clinical Oncology ‘‘5 per mille’’ to G.D.S. Funding to pay the Open Access publication charges for this article was provided by The Fondazione Telethon Italy.

## Supplementary Material

Supplementary Data
