# Supplementary material for: DLX5, FGF8 and the Pin1 isomerase control ΔNp63α protein stability during limb development: a regulatory loop at the basis of the SHFM and EEC congenital malformations
Source: Hum Mol Genet. 2014 Feb 25;23(14):3830–42. doi: 10.1093/hmg/ddu096 (PMC4065156; doi:10.1093/hmg/ddu096)
Supplement: Supplementary Data [file supp_23_14_3830__index.html]

DLX5, FGF8 and the Pin1 isomerase control ΔNp63α protein stability during limb development: a regulatory loop at the basis of the SHFM and EEC congenital malformations — DLX5, FGF8 and the Pin1 isomerase control ΔNp63α protein stability during limb development: a regulatory loop at the basis of the SHFM and EEC congenital malformations — DLX5, FGF8 and the Pin1 isomerase control ΔNp63α protein stability during limb development: a regulatory loop at the basis of the SHFM and EEC congenital malformations — DLX5, FGF8 and the Pin1 isomerase control ΔNp63α protein stability during limb development: a regulatory loop at the basis of the SHFM and EEC congenital malformations — Supplementary Data 

# *DLX5, FGF8* and the *Pin1* isomerase control ΔNp63α protein stability during limb development: a regulatory loop at the basis of the SHFM and EEC congenital malformations

## Supplementary Data

Supplementary Data

**Files in this Data Supplement:**

- Supplementary Data - Doc file
