# Supplementary material for: DLX5, FGF8 and the Pin1 isomerase control ΔNp63α protein stability during limb development: a regulatory loop at the basis of the SHFM and EEC congenital malformations
Source: Hum Mol Genet. 2014 Feb 25;23(14):3830–42. doi: 10.1093/hmg/ddu096 (PMC4065156; doi:10.1093/hmg/ddu096)
Supplement: Supplementary Data [file supp_ddu096_ddu096supp.doc]

**Restelli at al.**

***DLX5, FGF8* and the *Pin1* isomerase control ΔNp63α protein stability during limb development: a regulatory loop at the basis of the SHFM and EEC congenital malformations**

Michela Restelli, Teresa Lopardo, Nadia Lo Iacono, Giulia Garaffo, Daniele Conte, Alessandra Rustighi, Marco Napoli, Giannino Del Sal, David Perez-Morga, Antonio Costanzo, Giorgio Roberto Merlo and Luisa Guerrini

**Supplementary Figures and Table**

**
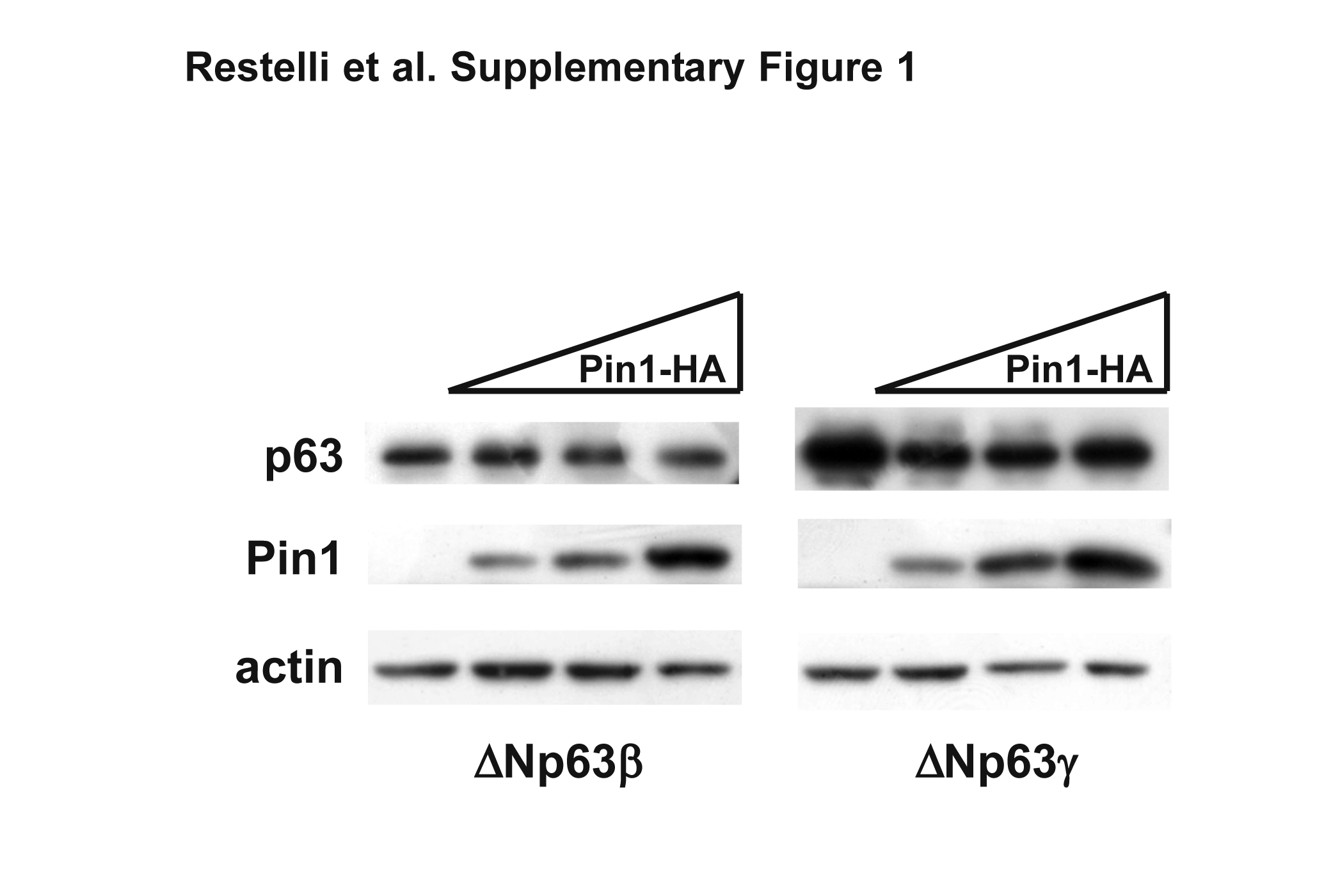
**

**Legend to Supplementary Figure 2**

Western blot analysis of whole protein extracts from U2OS cells transiently co-transfected with increasing amounts (20, 40 and 80 ng) of *Pin1*-*HA*-tagged vector (indicated on top), and wild-type ΔNp63β (on the left) or ΔNp63γ (on the right) (30 ng each). As opposed to the α isoform, the β and γ isoforms are not degraded by *Pin1* expression.

**
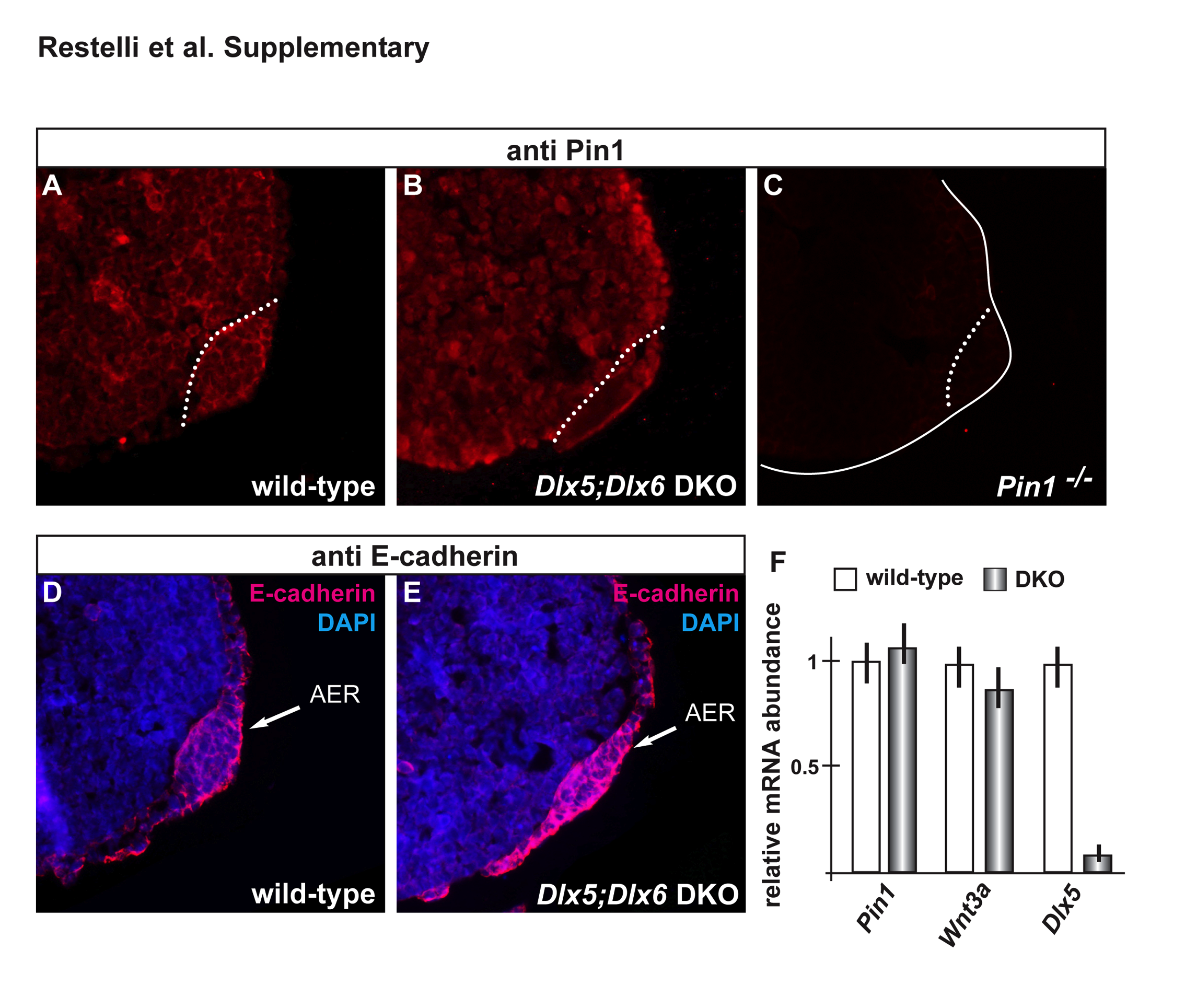
Legend to Supplementary Figure 2**

**A,B.** Immunofluorescent staining with anti-Pin1 on sections of the embryonic HLs of wild-type (A) or *Dlx5;Dlx6*-/- (B) embryos, at the age E11. Scale bar in A=20 μm. **C.** As control, the same staining was done on sections of HLs from *Pin1* KO embryos, at the age E11. The lack of staining in the *Pin1*-/- specimen indicates that the antibody is highly specific. **D,E.** Staining with anti-E-cadherin on adjacent sections, counterstained with DAPI. The AER is outlined with a dotted line and indicated with white arrows. **F.** Real-Time quantitative determination of the relative abundance of the mRNA of *Pin1, Wnt3a* and *Dlx5* in RNA samples from the HLs of wild-type and *Dlx5;Dlx6*-/- embryos, normalized against *GAPDH* and *TBP* mRNA. The abundance of the wild-type is set=1. As expected, the *Dlx5* mRNA is nearly absent. The *Pin1* mRNA is slightly increased while the *Wnt3a* mRNA is slightly decreased, however these differences are not significant.

**
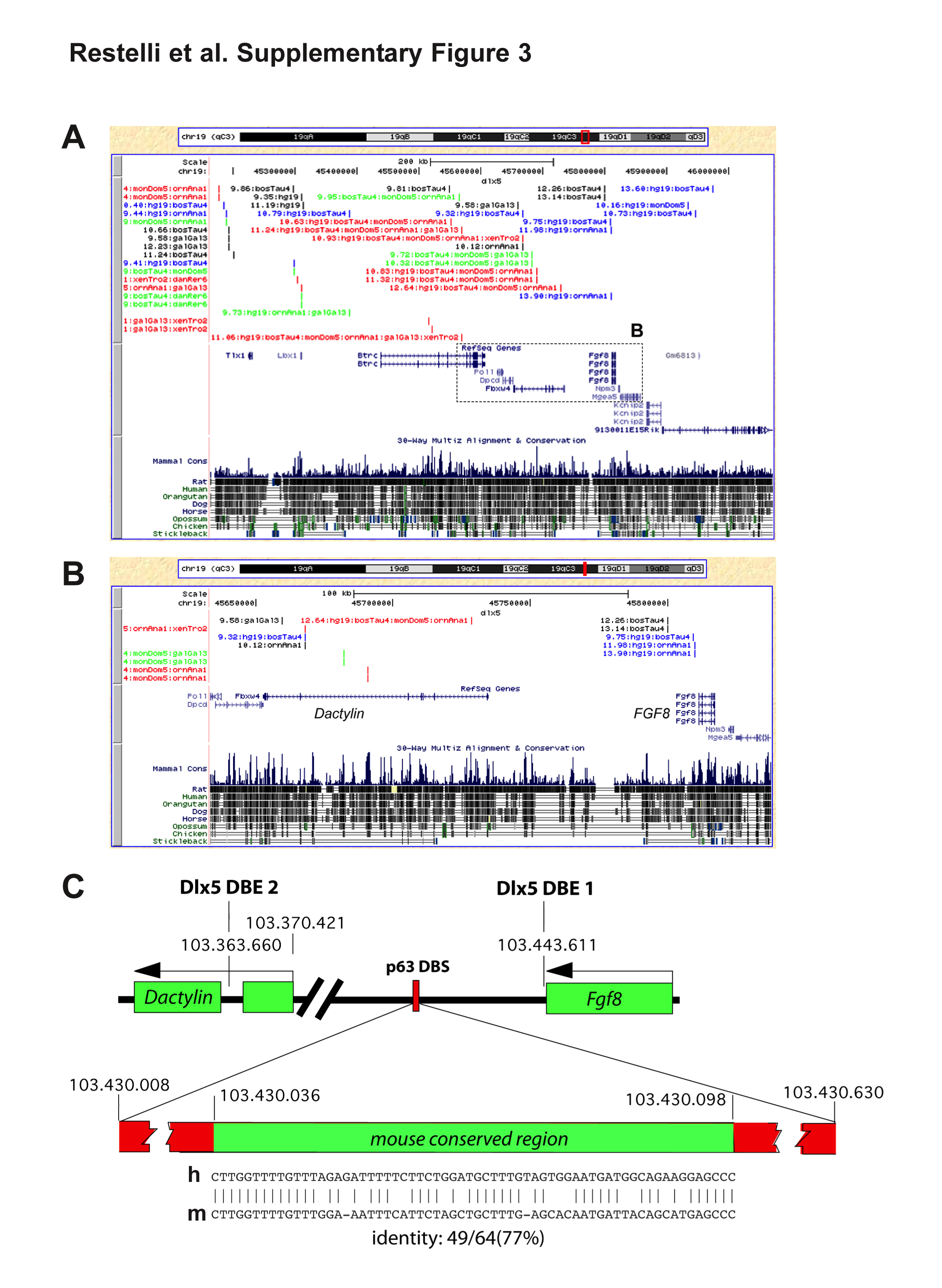
**

**A.** Location of predicted conserved Dlx5 binding elements (DBE) around the around the murine *Dactylyn* and the *FGF8* loci on chromosome 19q (10q in human), based on the UCSC mouse genome browser. Dlx5 sites were bio-informatically predicted using the published PWM, as described (ref. (31)). Sites are indicated with coloured vertical bars (asterisk) and annotated with the species conservation. A color code is used to indicate the number of species in which the site is conserved. Red indicated the most conserved ones. **B.** Enlargement of the area indicated with a solid box in A, centred around the genomic region comprising *FGF8* and *Dactylyn*. The chromosomal position and coordinates are reported on the top, the mammalian genomic conservation is reported on the bottom. **C. (top)** Location of the DLX5 DBE-1 and DBE-2 sites, corresponding to the sites tested by ChIP analysis (see Fig. 5 and corresponding text), in the human genomic region around *FGF8* and *DACTYLYN* (chrom. 10q). **(bottom)** Location of a p63-binding site (p63 DBS) within a conserved region of the human genome, between *FGF8* and *DACTYLYN*, as reported (ref. (25)).

**Supplementary Table I**

**A.** Sequences of the primers used for Real-Time qPCR on mouse embryonic tissues.

*mGAPDH* F 5’ TGTCAGCAATGCATCCTGCA

*mGAPDH* R 5’ TGTATGCAGGGATGATGTTC

*mTBP* F 5’ GGGTTATCTTCACACACCATGA

*mTBP* R 5’ CGGTCGCGTCATTTTCTC

*mRps9* F 5’ GACCAGGAGCTAAAGTTGATTGGA

*mRps9* R 5’ TCTTGGCCAGGGTAAACTTGA

*mDlx5* F 5’ TCTTATGGCAAAGCGCTCAA

*mDlx5* R 5’ CGTTCACGCCGTGGTACTG

*mDlx6* F 5’ TCCAGTGTGGGACGTTTCTG

*mDlx6* R 5’ CTGTTGGGAGGCATACTGACG

*mFGF8* F 5’ TGAGCTGATCCGTCACCA

*mFGF8* R 5’ TCCTGCCTAAAGTCACACAGC

*mPin1* F 5’ GTCCCTTCAGCAGAGGTCAG

*mPin1* R 5’ ACAGTAGCAGGAAGGGCATC

*mWnt3a* F 5’ GAGTGCTCAGAGAGGAGTACTGG

*mWnt3a* R 5’ CTTAGTGCTCTGCAGCCTGA

*mΔNp63* F 5’ ATGTTGTACCTGGAAAACAATG

*mΔNp63* R 5’ GATGGAGAGAGGGCATCAAA

**B.** Sequences of the oligonucleotides used for ChIP analysis on Dlx5 Binding Elements (DBE) near the *FGF8* locus

DBE-1 (FGF8) For CCTCTGAAGACTCGGATGTTCC

DBE-1 (FGF8) Rev AGGAAACGCTTTCATCTGCAC

DBE-2 (FGF8) For GCCCGAGGCAGCTTGTCTA

DBE-2 (FGF8) Rev GAGCCCTCACTAATGGGGTTTTA
